# Evaluation of follicular fluid's Beta-Human chorionic gonadotropin in the follicles of patient undergoing Intracytoplasmic sperm injection: A cross-sectional study

**DOI:** 10.18502/ijrm.v16i12.3686

**Published:** 2019-01-28

**Authors:** Masoumeh Hajshafiha, Tahere Behrouzi lak, Nasrin Hajiloo, Yaghoub Deldar, Mina Ghorbani, Fedyeh Haghollahi

**Affiliations:** ^1^Department of Obstetrics and Gynecology, Urmia Reproductive Health Research Center, Urmia University of Medical Sciences, Urmia, Iran.; ^2^Motahary Hospital, Urmia University of Medical Sciences, Urmia, Iran.; ^3^Clinical Biochemistry, Urmia University of Medical Sciences, Urmia, Iran.; ^4^Vali-Asr Reproductive Health Research Center, Tehran University of Medical Sciences, Tehran, Iran.

**Keywords:** * Intracytoplasmic sperm injection*, * Empty follicle syndrome*, * HCG.*

## Abstract

**Background:**

The failure to retrieve oocytes from mature ovarian follicles is referred to as empty follicle syndrome. There is no exact explanation to this problem and it cannot be predicted using ultrasound or serum hormonal levels. The underlying mechanism of Empty follicle syndrome remains obscure.

**Objective:**

In this study, the authors have investigated the relationship between the Beta-Human chorionic gonadotropin (βHCG) levels in the follicular fluid with or without the oocyte in the follicles of patients undergoing Intracytoplasmic Sperm Injection.

**Materials and Methods:**

Seventy-three infertile couples underwent standard long protocol induction ovulation for Intracytoplasmic sperm injection. On the day of oocyte retrieval, each patient had two samples; follicular fluid including 2–3 follicles with oocyte and follicular fluid including of 2–3 follicles without oocyte were collected in separate tubes. These follicles had similar shape and size. The Samples were transferred to a laboratory for measuring the βHCG level, after which the βHCG levels were compared to the follicles with and without the oocyte in each patient.

**Results:**

In this study, the βHCG level of follicular fluid in the follicles containing oocyte was 18.20 (8.35–42.92) IU/L and in the follicles without the oocyte was 13.50 (5.45–25.81) IU/L. Levels of βHCG in the follicular fluids containing the oocyte were higher than without oocytes, This difference was not statistically significant (*p* = 0.16).

**Conclusion:**

It seems that the follicular fluid βHCG isn't caused by empty follicle syndrome, and that dysfunctional folliculogenesis may be the cause of this syndrome.

## 1. Introduction

Empty follicle syndrome (EFS) is a disorder in in vitro fertilization cycles; no oocytes are retrieved from the mature follicle after ovulation induction (1). The hypothesis of this disorder is under debate (2, 3). The occurrence of this syndrome has been estimated to vary between 0.045 and 7% (3, 4). This variation may be due to different inclusion criteria (2). Stevenson and Lashen defined two types of EFS in 2008 (5). The beta-human chorionic gonadotropin (βhCG) level at the time of oocyte retrieval was the base on their description. The authors described that one type of EFS showed βhCG levels below optimal, while the other type showed the optimal βhCG levels. The optimal level of βhCG on the day of the follicular puncture was ≥ 40 mIU/mL. The mechanism responsible for this syndrome remains unclear (6, 7).

However, some believe that early oocyte atresia due to dysfunctional folliculogenesis is one cause of this syndrome (8). A longer contact with hCG, ovarian aging in older women (9), genetic factors, low bioavailability of hCG (10–12), a decrease in estradiol levels before the hCG injection (2, 13), rapid metabolic clearance, intrinsic problems of the drug, and human error (14) are other causes of empty follicle cycles in which no oocytes are retrieved. The Further review study in 2012 reported that In spite of a satisfactory ovarian response and normal level of hCG, no oocytes aspirated (14). Also, it was reported in other studies that the low-level of hCG caused the EFS (15–20). This syndrome leads to psychological and physical trauma in the patients. Due to the lack of a comprehensive view of the factors involved in this syndrome and the importance of this phenomenon in assisted reproductive techniques, we decided to examine, whether the β-hCG in the follicular fluid can be a marker for determining the existence or absence of oocytes in a follicle.

Therefore, we aimed to compare the B-hCG in the follicular fluid with or without oocytes in the follicle.

## 2. Materials and Methods

A cross-sectional study was performed in the infertility center of Shahid Motahary Hospital in Urmia, Iran, between May 2013 and September 2013. Seventy three infertile woman undergoing Intra Cytoplasmic Sperm Injection (ICSI) were included. The infertile women with age > 40 yr, hydro salpinx, endometriosis, follicle-stimulating hormone (FSH) >9 IU/L in the third day of the present menstrual cycle were excluded from the study. The stimulation of long Agonist protocol was mainly used for the induction to follicular growth.

In the agonist protocol, from the 21${}^{st}$ day of the previous cycle, Superfact (0.5 mg, S. c) was administrated and on the second day of the menstrual cycle, recombinant FSH (Gonal-F, Serono, Switzerland) (75 IU/ ampoule) was started, and continued depending on patient's response till the follicles size reached to 18 mm. Follicular growth was monitored by using the transvaginal sonography.

After the follicular diameter in 6–7 follicles reached 18–20 mm, 10,000 IU of human chorionic gonadotropin ((PregnylⓇ, Laboratories' Serono S.A.) was administered, and so then the follicular puncture was performed in the sterile condition later 34–36 hr after the hCG injection with the needle puncture (Double-lumen follicle aspiration needle, Cook catheter no. 17, COOK (CANADA) INC) by the same gynecologist. Six cc of follicular fluid including a minimum of 2–3 follicles (with or without oocyte) were gathered in tubes and sent to the same laboratory where they were assessed on two occasions in the presence or absence of the oocytes. Then, the same follicle was washed with 2–3 mL of the ringer, and the Follicular fluid obtained from washing was assessed for the presence of oocyte. The Fluid before and after washing was unmixed. If there was no oocyte after follicular washing, the first follicular fluid was considered as a follicular fluid without oocytes. If there was oocyte after washing, the first follicular fluid was considered as a Follicular fluid containing the oocyte. Tubes of follicular fluid were labeled in one (with oocyte) or two (without oocyte), β-hCG Test assessed on two occasions by the chemiluminescent assay (Liaison Kitt, Diasorin LTD, Italy). The β-hCG levels in the follicular fluid were compared in the two occasions of with or without of oocyte.

The patient was excluded if there were oocytes in all follicles or no oocyte in all follicles or oocyte in less than two obtained follicles, as it was not enough to assess the B-hCG level. Also, the oocyte was excluded if it was not in the stage of metaphase II.

### Ethical consideration

This study was approved by the Ethics Committee of the Urmia University of Medical Sciences (Code:IR.UMSU.reec.1393.214), and after providing the necessary explanations to the patients, the informed written consent was taken from each patient.

### Statistical analysis

After collecting the required information, the data were analyzed using SPSS software (Statistical Package for the Social Sciences, version 20.0, SPSS Inc, Chicago, Illinois, USA). Frequency and frequency percentage were calculated for qualitative variables. Means and standard deviations were determined for quantitative variables. Independent *t*-test, Chi-square test was used in the field. The significance level was considered as *p* < 0.05 to interpret the relationships among the variables. Distribution of β-hCG in this study was not normal distribution through which median and quartile were obtained, and the difference in two groups was assessed by the Mann–Whitney test, and then the *p*-value was extracted.

## 3. Results

In this cross-sectional study, 73 infertile patients aged between 20 and 40 years, candidate to ICSI and with an average age of 30.61 ± 5.4 yr and duration of infertility of 6.84 ± 4.3 yr were studied. The most common cause of infertility was the male factor (56.6%). Demographic characteristics are shown in Table I.

The level of β-hCG in the follicles containing the oocyte was higher than the follicles without oocytes, but there was no significant difference in β-hCG levels in the follicular fluid of patients with or without oocyte (*p* = 0.16) (Table II).

Considering that the aim of this study is to evaluate the β-hCG level in the follicular fluid with or without oocyte in any age, we wanted to know the association of the number of the oocyte, FSH, and β-hCG of follicular fluid with the oocytes in the age group under 38 or over 38 yr of age. The results indicate that the number of oocytes is lower in less than 38 yr, but the levels of β-hCG in follicles with or without oocytes are no different, that is, this is not related to the age. (Table III).

**Table 1:** Baseline characteristics in patients.


**Variable**		**N = 73**
Age (years)*		30.61 ± 5.4
Duration of infertility (years)*		6.84 ± 4.3
FSH of third day (Iu/l)*		5.56 ± 4.71
Endometrial Thickness (MM)*		9.86 ± 1.66
Nom of oocyte*		8.30 ± 4.30
Cause of infertility**		
	–male factor	41 (56.6)
	–Female factor	8 (11.5)
	–Unknown	24 (31.9)

Note: *data presented as Mean ± SD; *T*-Test;

** Data presented as n (%). Chi-square Test.

**Table 2:** Mean of β-hCG level in a follicular fluid with and without oocytes.


**Variable**	**Follicle without oocyte**	**Follicle with oocyte**	**p-value**
	**Median (Q1 – Q4)**		
β-hCG (IU/L)	13.50 (5.45–25.81)	18.20 (8.35–42.92)	0.16
Number of follicles	73	73	

Note: *Mann–Whitney test;

βhCG: Beta-human chorionic gonadotropin.

**Table 3:** The relationship of variables with follicular fluid (with or without the oocyte) in two age groups.


	**< 38 yr**	**38 yr ≤**	**p-value**
	**Mean ± SD**		
FSH of third day (Iu/l)*	4.2 ± 2.1	7.2 ± 2.6	< 0.001
No of oocyte	7.6 ± 1.2	5.1 ± 0.9	< 0.001
Median (Q1–Q3)			
β-hCG levels in the follicular fluid with oocyte	16.16 (4.95–41.87)	27.24 (12.47–47.50)	0.11*
β-hCG levels in the follicular fluid without oocyte	11.88 (2.75–23.34)	19.68 (1.030–35.54)	0.09*
N	58	15	

Note: *Mann–Whitney U test.

## 4. Discussion

This study showed that the levels of β-hCG in follicles with or without oocytes are no different.

To our knowledge, this is the first published report examining β-hCG levels in the follicular fluid with and without the oocyte in one person's follicles. This novel observation particularly reveals that although the level of β-hCG in the follicles containing oocyte was higher than the follicles without oocytes, there were no significant differences in β-hCG levels in follicular fluid in the presence or absence of oocyte in patients. It should be noted that in the current study, both follicles with oocytes and without oocytes were examined in one person.

Therefore, the HCG doses and hepatic clearance, the medical errors, the type of hCG consumed and the level of estradiol and progesterone are the same. Therefore, according to the results of the present study, hCG does not seem to play a role in the EFS, and folliculogenesis disorders in each follicle can be the cause of this phenomenon which is consistent with the Desai, Zreik studies (19, 20).

But Hassan and colleagues and Christopoulos' studies showed that in a variant of EFS, the cycle could be recovered by giving another dose of hCG (17, 18). Also, Ndukwe suggested in 2012 that time and dose of hCG can be the cause of this syndrome (11). Also, the optimal prognosis of these patients is still poorly understood. Large, systematic multi-center studies are needed to increase the understanding of EFS (3).

Therefore, In order to minimize the risk of folliculogenesis disorder and increase the probability of the existence of oocyte. It is recommended that it would be designed for basic studies to identify the critical markers involved in this syndrome. Conclusions: Our results show that the follicular fluids βhCG aren't caused by EFS; dysfunctional folliculogenesis may be the cause of this syndrome.

## 5. Limitation

The low sample size and the lack of a similar study to compare with our study are the limitations of our study.

##  Conflict of Interest

It should be noted that there was no association between the authors and any organization or institution. The authors report no declarations of interest.
